# Supplementary material for: Uncovering the viral aetiology of undiagnosed acute febrile illness in Uganda using metagenomic sequencing
Source: Nat Commun. 2025 Mar 23;16:2844. doi: 10.1038/s41467-025-57696-8 (PMC11930947; doi:10.1038/s41467-025-57696-8)
Supplement: Supplementary file 1 — Supplementary Information [file 41467_2025_57696_MOESM1_ESM.pdf]

Supplementary Information

Uncovering the viral aetiology of undiagnosed acute febrile illness in Uganda using metagenomic sequencing

(a)

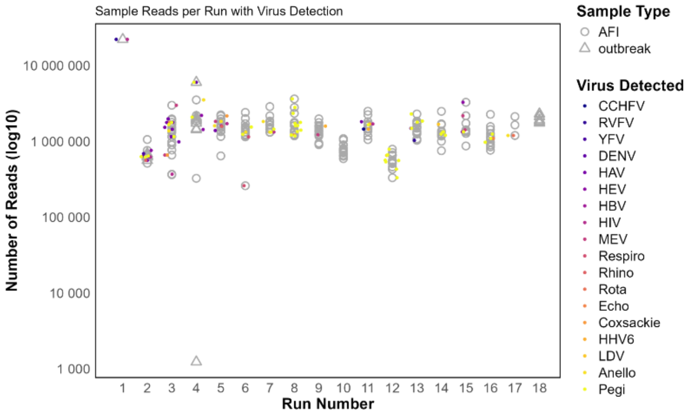

(b)

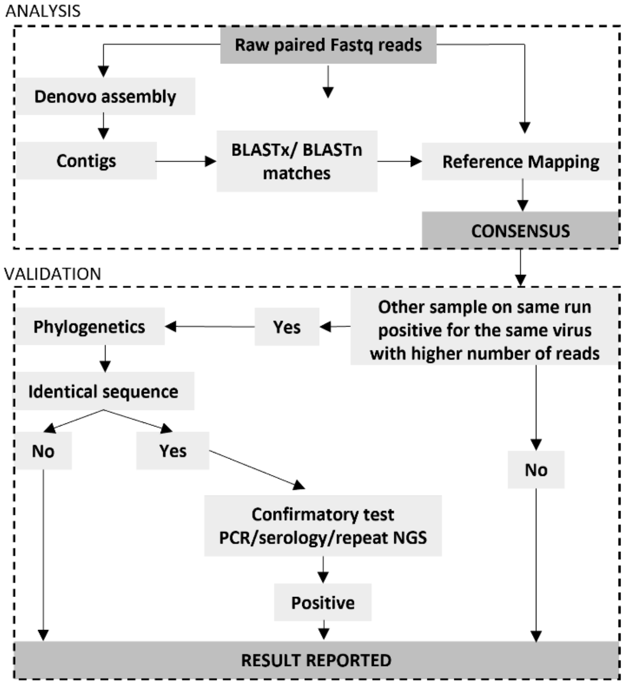

(c)

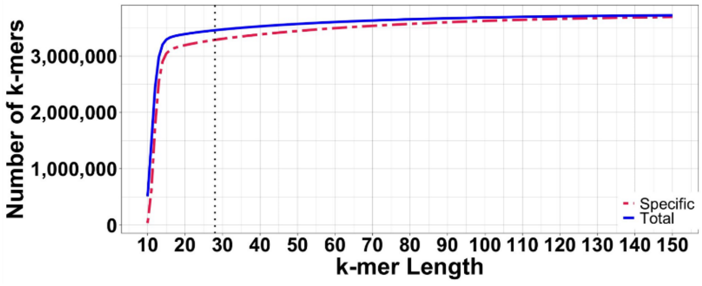

Supplementary Figure 1. Sequencing characterisation and analysis (a) Sequencing details showing samples multiplexed on each run with total reads obtained on y-axis for samples (shown as circles and triangles). Each sample icon also shows virus(es) detected in that sample. **(b)** Bioinformatics analysis pipeline. **(c)** Plot showing total and specific k-mers from 129 human viral pathogens (https://viralzone.expasy.org/678). K-mers of size 10-150bp were generated and counted from 129 viral genomes (red). Specific k-mers, i.e unique to a single genome in the dataset are shown in green. More than 95% of the total k-mers become specific to a sequence with k size of 28bp (dotted line) or more.

Identification of a novel rhabdovirus in blood tubes (Adumi virus)

A genome of 11613 base pairs containing five open-reading frames corresponding to the N, P, M, G and L genes (**Supplementary figure 2a**) of the *Ohlsravirus* genus of mosquito rhabdoviruses was detected in seven samples. Phylogenetic association and paiwise amino acid distances with related members of *Rhabdoviridae* is shown in (**Supplementary figure 2c,d**).This was considered most likely to be a contaminant of blood tubes, as nearly identical genomes were identified in all seven samples, obtained from geographically distant sites and we were unable to detect seroconversion in any of these patients (**Supplementary figure 2b**). Real-time PCR and re-extraction of RNA from original samples repeatedly tested positive. Reads from mosquito genomes were also detected in the positive samples, suggesting source contamination of blood collection tubes by mosquito-related material. In the absence of sufficient supporting evidence and because of its relatedness to viruses found in mosquitoes, we suspect this to have been a sporadic contaminant present in blood bottles.

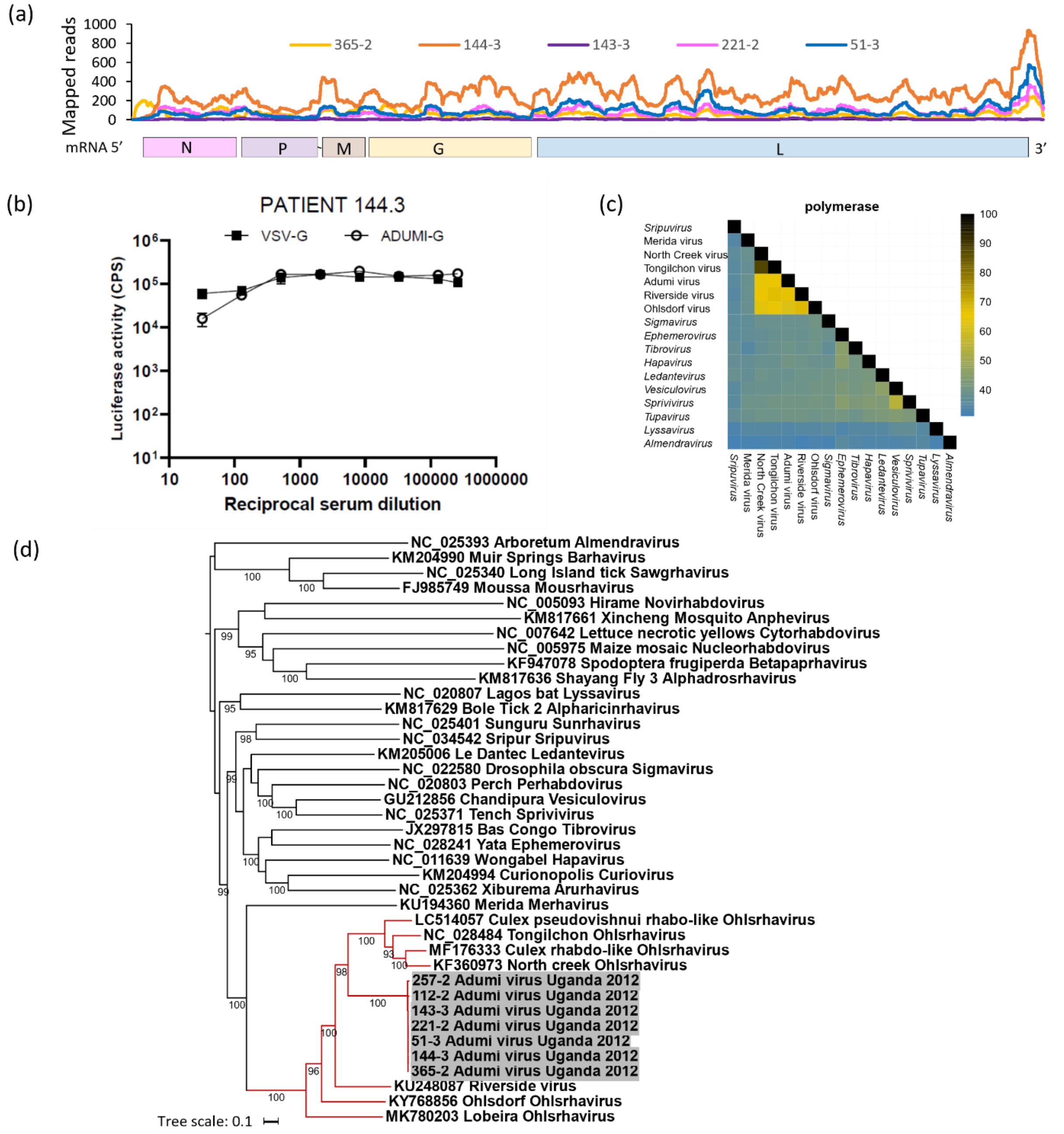

Supplementary Figure 2. Adumi virus identified from AFI patient samples. (a) Coverage plot showing depth (y-axis) and coverage across genome (x-axis) for five patient samples where the full genome of Adumi virus was recovered. Two patients (112-2, 257-2) where only partial genomes were found are not shown here. Genome structure is diagrammatically shown below the plot proteins represented as N=nucleoprotein, P=phosphoprotein, M=matrix, G=glycoprotein, L=polymerase. **(b)** Pseudotype neutralisation assay showing luciferase reporter activity (y-axis) against increasing serum dilutions (x-axis) for patient 144-3 using Adumi pseudotype virus compared with VSV control. Data points represent mean and SEM for n=3 technical replicates for one representative experiment. **(c)** Heat map showing pairwise amino acid percentage similarity for Adumi virus polymerase against selected rhabdovirus sequences, generated using MEGA 10 . **(d)** Phylogenetic full genome nucleotide tree using GTR+F+R4 model for *Rhabdoviridae* sequences. One sequence per genus was selected. In addition, all full genomes for *Ohlsrhavirus* were added (red branches). Tip labels represent virus and genus name. Evolutionary scale and ultrafast bootstrap values >90 are shown for nodes. Patients' samples are highlighted in grey.

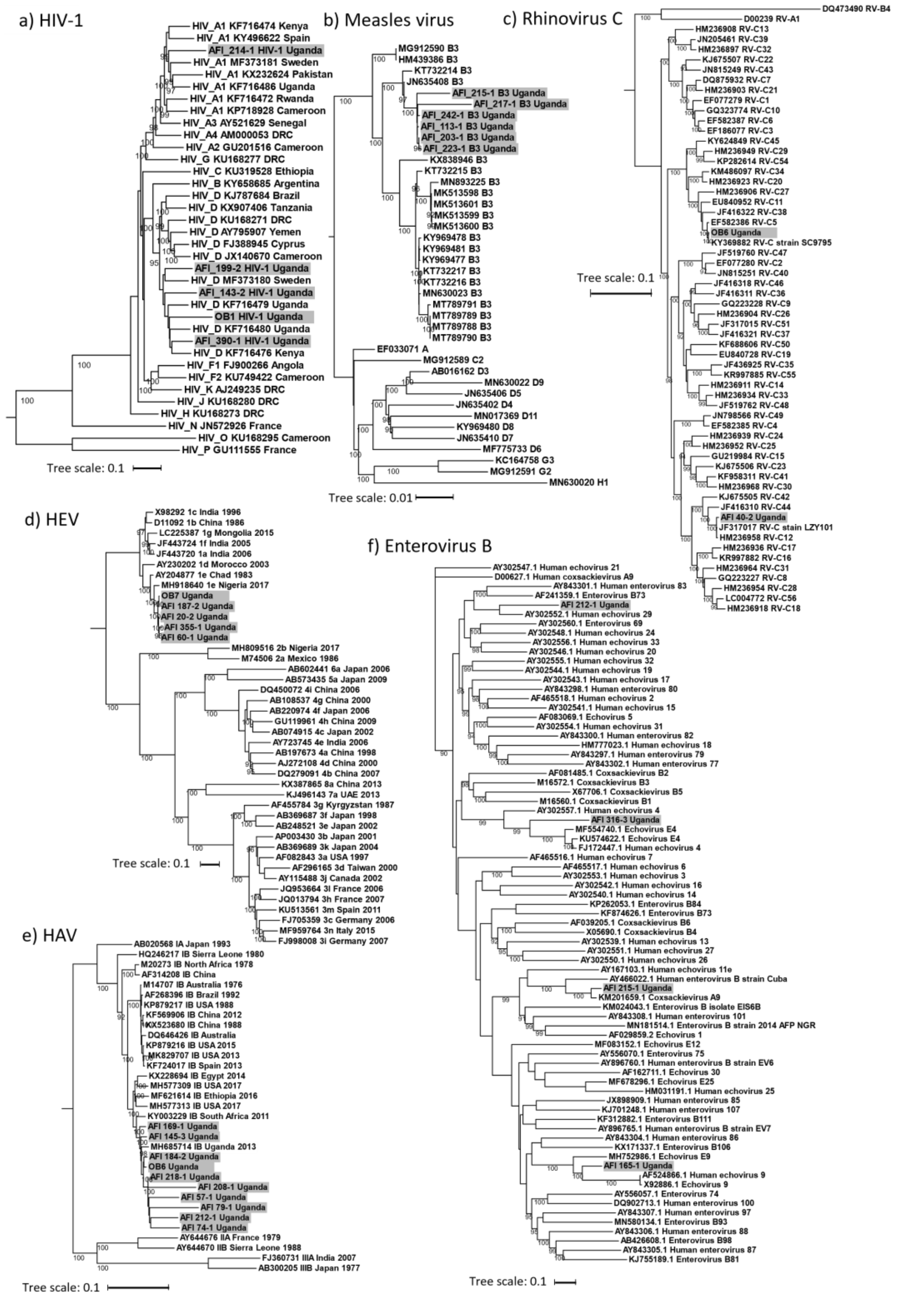

Supplementary figure 3. Phylogenetic trees for other known pathogens found in patients. Study samples with at least 10% genome coverage are included in phylogenetic trees. Evolutionary scale and ultrafast bootstrap values >90 are shown for nodes. Patients' samples are highlighted in grey. Relevant representative sequences are included for each clade on the tree and additonal sequences added for clades of interest to show clustering of AFI derived sequences. **(a)** HIV-1 tree showing groups M,N,O,P and subtypes of group M (A1-K) created using model GTR+F+R6. **(b)** Measles morbillivirus tree showing genotypes A-H1 created using model TIM+F+G4. **(c)** Rhinovirus C tree showing representative sequences for genotypes of RV-C and also one sequence of RV-A and RV-B created using model GTR+F+I+G4. **(d)** HEV tree showing genotypes 1a-8a with country and year of isolation, created using model GTR+F+R4. **(e)** HAV tree showing genotypes IA-IIIB with country and year of isolation, created using model TIM2+F+G4. **(f)** Enterovirus B tree showing genotypes for which full genomes were available, created using model GTR+F+R6.

Supplementary methods

Patients and sampling

1281 patients were recruited prospectively with informed consent into the AFI study from three study sites in Uganda; Ndejje HC IV (Wakiso), St. Paul's HC IV (Kasese), and Adumi HC IV (Arua) between April 2011 and January 2013 (**Figure 1a**). Inclusion criteria were being >=2 years age with (a) a fever lasting 2-7 days or ≥38°C temperature on admission, or (b) symptoms consistent with brucellosis or typhoid fever, as previously described. Cases with clinical evidence of an alternative diagnoses such as otitis media were excluded. Samples were obtained at presentation (acute sample) and 14-21 days later (convalescent sample). Diagnostic assays including serology, blood culture and blood films were carried out to identify and exclude active or recent infection with malaria, typhoid, leptospirosis, rickettsiae, CHIKV, dengue (DENV), WNV, YFV and ONNV. 210 patients remained undiagnosed and plasma from these patients were retrospectively tested using mNGS. In addition, 20 samples obtained from cases during suspected viral outbreaks referred to diagnostic services at UVRI between April 2013 and July 2016 were also included. As part of UVRI diagnostic testing, samples were tested for YFV, WNV, DENV, ONNV by PCR and for YFV, WNV, DENV, ZIKV, CHIKV by IgM as part of yellow fever virus outbreak surveillance (**Supplementary data 3**). Three of the samples were additionally tested for EBOV, MARV, CCHFV as part of VHF surveillance and one for RVFV.

Inclusion and Ethics statement

Ethical approval for the AFI study was granted by the UVRI Research Ethics Committee (GC/127/10/02/19) and the Uganda National Council for Science and Technology (HS767). Participation was unselected (allcomers).

Statistical analysis

Clinical, environmental and socioeconomic demographic variables recorded for 1281 AFI patients were subjected to a contingency analysis for the outcome of positive/negative viral infection determined through a combination of Phase I diagnostic screening and Phase II mNGS. Univariable analysis was performed through Chi-squared or Fisher's exact tests for all categorical variables and t-test or Mann Whitney for continuous variables depending on the distribution and variability of the data. Variables reaching statistical significance ($p<0.05$) were included in a

stepwise multivariable analysis. All analyses were performed in RStudio 2024.12.0 Build 467 using R 4.4.2 and CRAN packages dplyr, MASS, pROC, readxl, epitools, DescTools,and gglplot2.

Sample processing and mNGS

RNA was extracted from 200µl of plasma using the Agencourt RNAdvance Blood Kit (Beckman Coulter), according to manufacturer's instructions, including DNase treatment at 37°C for 15min. RNA was reverse transcribed using Superscript III (Invitrogen) followed by dsDNA synthesis with NEBNext Ultra II Non-Directional RNA Second Strand Synthesis Module (New England Biolabs). Following dsDNA synthesis, libraries were prepared using LTP low-input Library preparation kit (KAPA Biosystems) as previously described. Resulting libraries were quantified with the Qubit 3.0 fluorometer (Invitrogen) and their size determined using a 4200 TapeStation (Agilent). Up to 21 libraries were pooled in an equimolar ratio for each run and sequenced on Illumina MiSeq platforms at UVRI and the MRC-University of Glasgow Centre for Virus Research, resulting in a median of 1.3 million read pairs per sample (**Supplementary figure 1a**). Repeat confirmatory sequencing was carried out on a subset of samples.

Bioinformatic analysis

Raw fastq files were searched directly for evidence of viral sequences using DIAMOND BLASTx **(Supplementary figure 1b)**. *De novo* assembly was carried out using dipSPAdes and contigs were identified using DIAMOND BLASTx against the nr database. Viral hits detected with BLASTx were confirmed with BLASTn and mapped to the closest reference genome using *Tanoti*, a mapper developed for analysis of highly diverse viral genomes (github.com/vbsreenu/Tanoti). If a virus was detected in multiple samples, minimum cross-contamination thresholds were used to confirm the presence of unique viruses in each sample, including (a) a read threshold of at least 10 reads when samples from the same run contained the same virus at >=50 reads, and (b) at least 50% of mapped reads were unique , and (c) these sequences did not cluster identically by phylogeny (using the scripts https://github.com/ecthomson/Contamination-Phylogeny and https://github.com/ecthomson/Contamination-Filter).

Taxonomic assignments were made using on the basis of a BLASTn e-score of at least 0.01 (an e-value of <0.01 represents the likelihood that a given alignment score (or better) would occur purely by random chance in <1% of cases). In an additional independent analysis using human viral pathogen genome sequences, we estimated that 28 continuous nucleotides is a reliable

minimum sequence length required for taxonomic assignment of viruses, which is well under the minimum sequence length in our samples (**Supplementary figure 1c**).

Multiple sequence alignments were generated using MAFFT v7.487 with local alignment and 1000 iterations. Maximum-likelihood phylogenetic analysis was carried out for viruses with at least 10 percent genome coverage using IQ-TREE multicore version 1.6.12 with 1000 ultra-fast bootstrap replicates using relevant reference sequences. Substitution models mentioned in figure legends were best fit models as reported by IQ-TREE. Tree annotations were performed in iTOL version 7 (https://itol.embl.de/). Uncorrected pairwise-distances were estimated using MEGA 10.0. Sequence read data is available under Bioproject PRJNA1143542.

Capture ELISA

Rhabdovirus (Le Dantec virus; LDV and Adumi virus) glycoprotein genes (synthesized by Eurofins Genomics) excluding the transmembrane regions (identified using TMHMM and TMpred) were amplified and cloned into the secretory mammalian expression vector pHLSec containing a C-terminal 6xHistidine tag. Human embryonic kidney cells (HEK 293T) were maintained in Dulbecco's modified Eagle's medium (DMEM) supplemented with 100IU/ml penicillin, 100µg/ml streptomycin, 2mM glutamine and 10% foetal bovine serum. Cells were transfected (Fugene 6, Promega) and cell supernatant containing secreted recombinant glycoproteins was harvested at 48-hours and expression confirmed by Western Blotting against a 6x histidine (His) tag. ELISA plates (ImmulonHB) were coated with rabbit anti-His antibody (1:1000, Abcam AB9108) overnight and blocked with 2.5% BSA/PBS at 37°C for 1 hour. Cell supernatant containing glycoprotein was added to the wells followed by patient serum, both for 1 hour at 37°C. This was followed by HRP-conjugated goat anti-human IgG (Sigma A0170, 1:60000) at room temperature for 1 hour. Wells were washed after each step with 0.1% Tween-20/PBS. Reactions were developed using TMB substrate, stopped with 0.16M sulphuric acid and read on a spectrophotometer (Pherastar).

Pseudotype neutralization assay

The use of vesicular stomatitis virus (VSV) in which the glycoprotein sequence has been replaced (VSVΔG*luc*) to generate pseudotyped viruses has been described previously. Glycoprotein genes were cloned into the eukaryotic expression vector and transfected into HEK-293T cells using polyethylenimine followed by infection with VSVΔG*luc*-VSV-G at a multiplicity of infection of

0.02. Cells were incubated for one hour at 37°C, washed with PBS and re-incubated in DMEM. Supernatants were harvested 72-hours post infection. Pseudoparticle activity was assessed on HEK-293T cells by measuring luciferase activity at 72-hours on a Microbeta 1450 Jet luminometer (Perkin Elmer). For neutralisation assays, 1×10^4 HEK-293T cells were plated in a white 96-well plate and incubated at 37°C for one hour. Four-fold serum dilutions were prepared in DMEM ranging from 1:8 to 1:131072. Serum was mixed with either VSVΔG*luc*-VSV-G or VSVΔG*luc*-LDV-G at a concentration expected to generate a luciferase reading of 1×10^6 counts per second and incubated for 1 hour at 37°C. 50µl per well of the pseudotype/serum mixture was added to the cells and incubated for 24 hours. Luciferase substrate was added to each well (Steadylite plus™, Perkin Elmer) and luminescence measured on a Chameleon V plate scintillation counter (Hidex Oy).
