# Supplementary material for: Uncovering the viral aetiology of undiagnosed acute febrile illness in Uganda using metagenomic sequencing
Source: Nat Commun. 2025 Mar 23;16:2844. doi: 10.1038/s41467-025-57696-8 (PMC11930947; doi:10.1038/s41467-025-57696-8)
Supplement: Supplementary file 2 — Description of Additional Supplementary Files [file 41467_2025_57696_MOESM2_ESM.pdf]

Description of Additional Supplementary Files

Supplementary Data 1. Details of viral genomes detected by mNGS with percentage coverage, reads mapped, nearest full genome reference sequence by blastn of consensus and percentage nucleotide (nt) identity. If the nearest blastn match was a partial genome, it is indicated in the table.

Supplementary Data 2. Human symbionts (HpGV and Anelloviruses; TTV, TTMV, TTMdV, TTV-like) found in patient samples showing percentage coverage and reads mapped for each sample.

Supplementary Data 3. Outbreak samples with test results for suspected viral infections.

Supplementary Data 4. Details of Univariate and Multivariate statistical analyis of 1281 patient AFI cohort. Data is presented as odds ratio, 95% confidence interval and p-values (Chi-squared and t-tests).
