# Supplementary material for: Uncovering the viral aetiology of undiagnosed acute febrile illness in Uganda using metagenomic sequencing
Source: Nat Commun. 2025 Mar 23;16:2844. doi: 10.1038/s41467-025-57696-8 (PMC11930947; doi:10.1038/s41467-025-57696-8)
Supplement: Supplementary file 7 — Reporting Summary [file 41467_2025_57696_MOESM7_ESM.pdf]

Corresponding author(s): Emma Thomson

Last updated by author(s): Feb 4, 2025

Reporting Summary

Nature Portfolio wishes to improve the reproducibility of the work that we publish. This form provides structure for consistency and transparency in reporting. For further information on Nature Portfolio policies, see our Editorial Policies and the Editorial Policy Checklist.

Statistics

For all statistical analyses, confirm that the following items are present in the figure legend, table legend, main text, or Methods section.

n/a	Confirmed	
☐	☒	The exact sample size (*n*) for each experimental group/condition, given as a discrete number and unit of measurement
☒	☐	A statement on whether measurements were taken from distinct samples or whether the same sample was measured repeatedly
☐	☒	The statistical test(s) used AND whether they are one- or two-sided<br>*Only common tests should be described solely by name; describe more complex techniques in the Methods section.*
☐	☒	A description of all covariates tested
☐	☒	A description of any assumptions or corrections, such as tests of normality and adjustment for multiple comparisons
☐	☒	A full description of the statistical parameters including central tendency (e.g. means) or other basic estimates (e.g. regression coefficient) AND variation (e.g. standard deviation) or associated estimates of uncertainty (e.g. confidence intervals)
☐	☒	For null hypothesis testing, the test statistic (e.g. *F*, *t*, *r*) with confidence intervals, effect sizes, degrees of freedom and *P* value noted<br>*Give P values as exact values whenever suitable.*
☒	☐	For Bayesian analysis, information on the choice of priors and Markov chain Monte Carlo settings
☒	☐	For hierarchical and complex designs, identification of the appropriate level for tests and full reporting of outcomes
☒	☐	Estimates of effect sizes (e.g. Cohen's *d*, Pearson's *r*), indicating how they were calculated

Our web collection on statistics for biologists contains articles on many of the points above.

Software and code

Policy information about availability of computer code

Data collection	No software was used for data collection
Data analysis	Raw fastq files were searched directly for evidence of viral sequences using diamond BLASTx. De novo assembly was carried out using dipspades and contigs were identified using diamond BLASTx against the nr database. Viral hits detected with BLASTx were confirmed with BLASTn (minimum escore of 0.01) and mapped to the closest reference genome using Tanoti, a mapper developed for analysis of highly diverse viral genomes (github.com/vbsreenu/Tanoti). Maximum-likelihood phylogenetic analysis was carried out for viruses with at least 10 percent genome coverage using IQTREE 1.6.12 and 1000 ultra-fast bootstrap replicates using relevant reference sequences. Uncorrected pairwise-distances were estimated using MEGA 10.0. If a virus was detected in multiple samples, minimum cross-contamination thresholds were used to confirm the presence of unique viruses in each sample, including (a) a read threshold of at least 10 reads when samples from the same run contained the same virus at >=50 reads, and (b) 50% of mapped reads were unique , and (c) these sequences did not cluster identically by phylogeny (using scripts https://github.com/ecthomson/Contamination-Phylogeny and https://github.com/ecthomson/Contamination-Filter).

For manuscripts utilizing custom algorithms or software that are central to the research but not yet described in published literature, software must be made available to editors and reviewers. We strongly encourage code deposition in a community repository (e.g. GitHub). See the Nature Portfolio guidelines for submitting code & software for further information.

Data

Policy information about availability of data

All manuscripts must include a data availability statement. This statement should provide the following information, where applicable:

- Accession codes, unique identifiers, or web links for publicly available datasets
- A description of any restrictions on data availability
- For clinical datasets or third party data, please ensure that the statement adheres to our policy

Sequence read data is available under Bioproject PRJNA1143542.Alignments and trees are available at https://github.com/ecthomson/AFI/.

Research involving human participants, their data, or biological material

Policy information about studies with human participants or human data. See also policy information about sex, gender (identity/presentation), and sexual orientation and race, ethnicity and racism.

Reporting on sex and gender	Recruitment was unselected and carried out on a first come first served basis based on clinic attendence with fever.
Reporting on race, ethnicity, or other socially relevant groupings	N/A
Population characteristics	1281 patients were recruited prospectively with informed consent into the AFI study from three study sites in Uganda; Ndejje HC IV (Wakiso), St. Paul's HC IV (Kasese), and Adumi HC IV (Arua) between April 2011 and January 2013.
Recruitment	Inclusion criteria were >2 years age with (a) a reported fever lasting 2-7 days or ≥38°C temperature on admission, or (b) symptoms consistent with brucellosis or typhoid fever, as previously described (Kigozi BK, Kharod GA, Bukenya H, Shadomy SV, Haberling DL, Stoddard RA, et al. Investigating the etiology of acute febrile illness: a prospective clinic-based study in Uganda. BMC Infect Dis. 2023;23(1):411). Cases with clinical evidence of an alternative diagnoses such as otitis media were excluded. Samples were obtained at presentation (acute sample) and 14-21 days later (convalescent sample). Diagnostic assays including serology, blood culture and blood films were carried out to identify and exclude active or recent infection with malaria, typhoid, leptospirosis, rickettsiae, CHIKV, dengue (DENV), WNV, YFV and ONNV. 210 patients remained undiagnosed and acute plasma were retrospectively tested using mNGS. In addition, 20 samples were obtained from two outbreaks of febrile illness that occurred within the time period of the study.
Ethics oversight	Ethical approval for the AFI study was granted by the Uganda Virus Research Institute (UVRI) Research Ethics Committee (GC/127/10/02/19) and the Uganda National Council for Science and Technology (HS767).

Note that full information on the approval of the study protocol must also be provided in the manuscript.

Field-specific reporting

Please select the one below that is the best fit for your research. If you are not sure, read the appropriate sections before making your selection.

☒ Life sciences ☐ Behavioural & social sciences ☐ Ecological, evolutionary & environmental sciences

For a reference copy of the document with all sections, see nature.com/documents/nr-reporting-summary-flat.pdf

Life sciences study design

All studies must disclose on these points even when the disclosure is negative.

Sample size	We used all available undiagnosed samples from the AFI study.
Data exclusions	Samples were excluded if they tested positive by serology or PCR for any pathogen prior to mNGS testing.
Replication	Data analysis of next generation sequencing data was carried out in parallel by two investigators and the results were compared. The presence of high consequence or emerging viruses was subsequently confirmed by PCR and/or serology where possible.
Randomization	NA
Blinding	Samples were anonymised and preceding symptoms or testing results were not known to the investigators prior to the analysis.

Reporting for specific materials, systems and methods

We require information from authors about some types of materials, experimental systems and methods used in many studies. Here, indicate whether each material, system or method listed is relevant to your study. If you are not sure if a list item applies to your research, read the appropriate section before selecting a response.

Materials & experimental systems

n/a	Involved in the study	
☐	☒	Antibodies
☐	☒	Eukaryotic cell lines
☒	☐	Palaeontology and archaeology
☒	☐	Animals and other organisms
☐	☒	Clinical data
☒	☐	Dual use research of concern
☒	☐	Plants

Methods

n/a	Involved in the study	
☒	☐	ChIP-seq
☒	☐	Flow cytometry
☒	☐	MRI-based neuroimaging

Antibodies

Antibodies used: goat anti-human IgG-HRP secondary antibody (Sigma A0170) 1:60000, Rabbit anti-6xHis antibody (Abcam AB9108) 1:1000

Validation: Validated for ELISAs

Eukaryotic cell lines

Policy information about cell lines and Sex and Gender in Research

Cell line source(s): HEK-293T cells used for production of recombinant antigen for ELISA and to produce pseudotyped virus for neutralisation assays.

Authentication: Not on the current ICLAC list

Mycoplasma contamination: Tested negative

Commonly misidentified lines (See ICLAC register): NA

Clinical data

Policy information about clinical studies

All manuscripts should comply with the ICMJE guidelines for publication of clinical research and a completed CONSORT checklist must be included with all submissions.

Clinical trial registration: NA

Study protocol: Not a clinical trial

Data collection: As previously described (Kigozi BK, Kharod GA, Bukenya H, Shadomy SV, Haberling DL, Stoddard RA, et al. Investigating the etiology of acute febrile illness: a prospective clinic-based study in Uganda. BMC Infect Dis. 2023;23(1):411)

Outcomes: As previously described (Kigozi BK, Kharod GA, Bukenya H, Shadomy SV, Haberling DL, Stoddard RA, et al. Investigating the etiology of acute febrile illness: a prospective clinic-based study in Uganda. BMC Infect Dis. 2023;23(1):411)

Plants

Seed stocks: NA

Novel plant genotypes: NA

Authentication: NA
